# Proteomic analysis of serum samples after cardiac arrest: Rationale and design of a TTM-trial substudy

**DOI:** 10.1016/j.resplu.2025.101014

**Published:** 2025-06-21

**Authors:** Gabriele Lileikyte, Anahita Bakochi, Marc Isaksson, Filip Årman, Marion Moseby-Knappe, Johan Malmström, Niklas Nielsen

**Affiliations:** a Department of Clinical Sciences Helsingborg, Anaesthesia and Intensive Care, Lund University, Skåne University Hospital, Malmö, Sweden; b Swedish National Infrastructure for Biological Mass Spectrometry (BioMS), Lund University, Lund, Sweden; c Department of Clinical Sciences Lund, Neurology and Rehabilitation, Lund University, Skåne University Hospital, Lund, Sweden; d Department of Clinical Sciences Lund, Infection Medicine, Lund University, Lund, Sweden; e Department of Clinical Sciences Lund, Anaesthesia and Intensive Care, Lund University, Helsingborg Hospital, Helsingborg, Sweden

**Keywords:** Out-of-hospital cardiac arrest, Heart arrest, Proteomics, Prognostication, Hypothermic temperature control, Targeted temperature management

## Abstract

**Background:**

A pilot study investigating proteomic profiles from 78 patients from the Target Temperature Management after Out-of-hospital Cardiac arrest (TTM) trial revealed 35 proteins associated to functional outcome, and six proteins associated to targeted temperature management at 33 °C. We present the protocol for a study investigating proteomic profiles in the full cohort of the TTM-trial biobank. The aim is to stratify protein profiles based on survival, functional outcome, targeted temperature management, and MIRACLE2 score in order to search for potential novel biomarkers.

**Methods:**

All patients with available serum samples at 24, 48, and/or 72 h after return of spontaneous circulation (*N* = 682 patients and *N* = 1882 samples) will be included in the liquid chromatography and tandem mass spectrometry analysis using *diaPASEF*, combining data-independent-acquisition of spectra with parallel accumulation-serial fragmentation. Statistical analysis will include data normalisation, exploratory principal component analysis, and differential expression analysis. Changes in serum protein abundance will be analysed according to survival and binary functional outcome (modified Rankin Scale 0–3 vs. 4–6) at six-months after randomisation, randomisation to target temperature of 33 °C or 36 °C, and the MIRACLE2 score. Secondary stratifications will include sex, age, time to return of spontaneous circulation, shockable vs. non-shockable initial rhythm, circulatory shock on admission, and presumed cause of death.

**Conclusion:**

This prospective study will provide information about proteomic profiles after cardiac arrest and may give insight for identification of novel biomarkers for prediction of outcome.

## Background

Proteomics represents the large-scale analysis of proteins that can be used for explorative discovery of temporal serum proteome profile differences between study groups.^1^ Such analysis has the potential to discover novel biomarkers and panels, as the unbiased nature of the analysis assists identification of proteins that might otherwise not have been considered relevant in the clinical situation. Currently, only serial measurements of the brain injury marker neuron-specific enolase are recommended for neuroprognostication of cardiac arrest survivors.^2^ Neurofilament light chain, total-tau, and glial fibrillary acidic protein are potential biomarkers not routinely in use for prediction of outcome after cardiac arrest.^2^, ^3^, ^4^, ^5^ Compared to other prognostication methods, biomarkers provide a quantitative result.^6^

Animal experimental data suggest that mild hypothermia, when applied prior to, during, or early after cardiac arrest, may exert neuroprotective effects through lowered cell metabolism, diminished excitotoxicity, reduced inflammation, modified gene expression, and anti-apoptosis.^7^ However, large clinical trials, such as the Target Temperature Management after out-of-hospital cardiac arrest (TTM) trial showed no clinical benefit of a target temperature of 33 °C as compared to 36 °C.^8^ Although targeted temperature management has been widely investigated in clinical and experimental setting, knowledge on how mild induced hypothermia affects the human body on protein level is limited.^9^, ^10^

We previously performed a pilot study to analyse proteomic profiles in serum samples from 78 out-of-hospital cardiac arrest (OHCA) patients included in the TTM-trial.^11^ Using data-independent acquisition mass spectrometry analysis, 403 proteins were confidently detected and quantified for statistical analysis comparing poor outcome to good outcome and targeted temperature management of 36–33 °C. Analysis of functional outcome yielded 35 statistically significant proteins involved in various biological processes, including apoptosis, inflammatory/immune responses, and cell activation. Evaluation of proteomic profiles according to temperature level revealed six statistically significant proteins mainly involved in inflammatory and immune responses.

This larger scale explorative study will aim to investigate proteomic profiles in cardiac arrest patients using liquid chromatography and tandem mass spectrometry (LC-MS/MS) analysis. Our aim is to investigate protein abundance stratified according to survival, functional outcome, targeted temperature management, and MIRACLE2 score to identify possible novel biomarkers. Secondary stratifications will include sex, age, shockable vs. non-shockable rhythm, circulatory shock on admission, and presumed cause of death.

## Methods

**Fig. 1:**
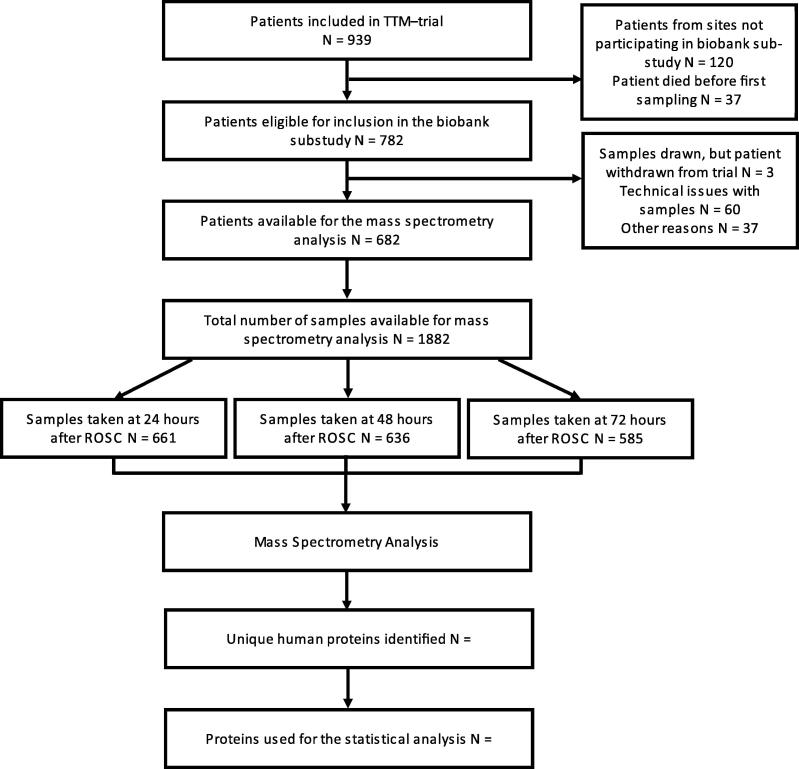
Example of patient flow-chart. **Abbreviations:** TTM, Target Temperature Management after Out-of-hospital Cardiac arrest trial; ROSC, return of spontaneous circulation.

All available serum samples collected during the TTM-trial (NCT01020916) will be included.^12^ The TTM-trial was a multicentre, international, randomised controlled trial including 939 patients with a presumed cardiac cause of out-of-hospital arrest.^8^ Patients were allocated to targeted temperature managements at 33 °C or 36 °C for 24 h as described.^13^ Serum samples were prospectively collected at 24, 48, and/or 72 h after return of spontaneous circulation (ROSC) and stored in a central biobank. A total of 682 unique patients with 1882 serum samples will be available for the LC-MS/MS analysis (Fig. 1). Processing of samples and LC-MS/MS analysis was carried out between 2023 and 2024. Additional in-depth analyses and data analyses are being conducted in 2025. This study has been registered at ClinicalTrials.gov (Identifier: NCT07017374).

### Outcomes

Patient data collected during the TTM-trial will be used for outcome evaluation and the subsequential statistical analyses.^8^, ^13^ Outcomes for the statistical analyses will include survival at 6 months, functional outcome, and targeted temperature management of 33 °C and 36 °C, as randomized in the TTM-trial.^8^ Functional outcome will be evaluated through the modified Rankin scale (mRS) determined during the TTM-trial by a face-to-face follow-up 6 months after OHCA.^14^, ^15^ The defined functional outcomes will be good outcome (mRS 0–3) vs. poor outcome (mRS 4–6).^16^ We also aim to evaluate proteomic profiles according to the MIRACLE2 score which was previously validated for assessing the severity and risk of poor outcomes following OHCA.^17^ Original MIRACLE2 publication stratified observed functional outcome by low, intermediate, or high admission MIRACLE2 score, defined as a score of 0–2, 3–4, or ≥5, respectively, with higher scores indicating a greater risk of poor outcome. We plan to compare low and intermediate admission score to high MIRACLE2 score, as our analysis requires a binary outcome. Calculation of MIRACLE2 score using TTM-trial data has been previously described.^18^

### Ethical approvals

The TTM-trial was approved by the ethical committees in each participating country (NCT01020916).^8^ This substudy is conducted under an approved amendment to the original ethical protocol, authorising the analysis of biological samples as described.

### Sample preparation for LC-MS/MS-analysis

All samples will be randomised prior to sample preparation to a randomised number ID. Ten microliter (μl) from each serum sample will be diluted manually into 1:10 with 100 mM ammonium bicarbonate (AmBic, Sigma-Aldrich, ReagentPlus®, ≥99%). From the diluted serum samples, 10 μl will be transferred to a 96-well plate and used for protein digestion. Samples will then be incubated for 60 min at 37 °C in 4 M urea (Sigma-Aldrich, ReagentPlus®, ≥99.5%) and 60 mM dithiothreitol (DTT, Sigma-Aldrich) for denaturation and reduction. Addition of DTT to the samples, as well as the following additions, will be performed with a BRAVO liquid handler (Agilent). The samples will then be alkylated using 80 mM 2-iodoacetamide (Sigma-Aldrich) for 30 min at room temperature in the dark. Primary digestion will be performed by addition of 100 μl LysC-protease (0.02 μg/μl, Lysyl Endopeptidase, Mass Spectrometry Grade, Wako) into each well and incubating the samples for two hours at room temperature. The samples will then be further digested using 100 μl trypsin (0.02 μg/μl, sequencing-grade modified porcine trypsin, Promega) for 16 h at room temperature. The digestion will be stopped manually by 10 μl 10% trifluoroacetic acid until pH ∼ 2. The samples will then be transferred to non-binding plates and covered with aluminium sealing tapes for subsequent storage in freezer (−80 °C) until LC-MS/MS analysis.

A constant volume of each sample will be loaded onto disposable Evotip C18 trap columns (Evosep Biosystems) according to the manufacturer’s instructions. The exact volume will be decided based on peptide or protein concentration determination of 92 randomized and prepared samples, and this volume will be implemented in all the consecutive samples. The expected load will range between 200 and 700 ng. Loading a constant volume instead of a fixed mass will better reflect protein abundances of clinical samples in the resulting mass spectrometry data. Briefly, the Evotips are activated in 0.1% formic acid in acetonitrile (solvent B, Supelco, acetonitrile with 0.1% (v/v) formic acid, hypergrade for LC-MS), conditioned by wetting the tips in 2-propanol (Honeywell, 2-propanol, ≥99.5%) and equilibrated in 0.1% formic acid (solvent A, Supelco, water with 0.1% (v/v) formic acid, hypergrade for LC-MS). Twenty μl sample is transferred to each tip, followed by washing the tips with solvent A. The Evotips are stored in solvent A before LC-MS/MS analysis.

### Enhancing proteomic depth in a subset serum analysis through enrichment techniques

Achieving comprehensive protein identification in serum and plasma remains a significant challenge due to the dynamic range of high and low-abundant proteins.^19^, ^20^ To address this, several enrichment strategies have been recently developed, such as ENRICHPlus (PreOmics) and P2 Plasma Enrichment System (Biognosys).^21^, ^22^ One of these techniques will be implemented in this study. ENRICHPlus technique involves bead-based enrichment of low-abundant proteins, followed by digestion and purification using paramagnetic beads.^21^ P2 Plasma Enrichment System utilizes a single-particle enrichment method combined with optimized surface chemistry and a novel buffer system to stabilise the labile protein corona formed around particles in blood.^22^ Both techniques enhance proteome coverage and reproducibility, enabling deeper protein identification. Enrichment of selected serum samples will be performed according to manufacturer’s instructions and publication of results will depend on whether the technique provides any new or valuable information.

### LC-MS/MS-analysis

#### diaPASEF runs

For all samples, the Evosep separation method of 60 samples per day (SPD) will be run on a timsTOF HT (Bruker Daltonics) mass spectrometer with a *diaPASEF* method, combining *data-independent-acquisition* (DIA) of spectra with *parallel accumulation-serial fragmentation* (PASEF).^23^ The Python package py_diAID^24^ will be used to automatically design variable isolation windows of the diaPASEF method over the selected mass-over-charge (*m*/*z*) range and ion mobility (IM) range. With this method, peptides in each sample are initially separated using the Evosep One liquid chromatography system prior to ionization and injection into the mass spectrometer.^25^ As peptides are ionized and injected into the instrument, they are separated based on their respective ion mobilities inside the *Trapped ion mobility spectrometer* (TIMS) module of the timsTOF instrument.^26^ Peptide ions are then released sequentially, as a function of their IM value, from the TIMS module for fragmentation and detection. In each cycle, the first spectra will be acquired in full scan mode (MS1) to record *m*/*z* intensities and IM values for precursor ions, followed by 12 acquisitions of diaPASEF spectra (MS2) to record *m*/*z* intensities and IM values for peptide fragment ions. For each diaPASEF scan, there will be two IM windows to cover the selected IM range.

#### ddaPASEF runs

Each prefractionated pooled sample will be run on the timsTOF HT with a ddaPASEF (DDA; data-dependent acquisition) method combined with the Evosep 60 SPD liquid chromatography method.

### Spectral library building

A deep, sample-specific spectral library will be built from acquired ddaPASEF runs using Fragpipe^27^, ^28^, ^29^, ^30^, ^31^ with the default spectral library workflow. In this workflow, the MSFragger search engine^27^ uses a sequence database to identify ddaPASEF MS2 spectra. Identified MS2 spectra, also referred to as peptide-spectrum-matches (PSMs), are then imported into Percolator^30^ for confidence estimation. Confident PSMs having a *q*-value ≤ 0.01 are loaded into the ProteinProphet algorithm,^31^ as implemented in the Philosopher^29^ toolkit, for grouping PSMs into proteins. PSMs of protein groups having a *q*-value ≤ 0.01 are subsequently passed to EasyPQP^32^ to build the spectral library.

### Data analysis

Acquired diaPASEF files (*.d-format) are automatically uploaded to a local openBIS^33^ server for annotation of MS-files, long-term storage and backup at Lund University Data Centre, Sweden. A dedicated *snakemake* workflow^34^ will be used to run all the necessary data analysis steps, such as downloading of files from the openBIS server to a workflow server, peptide-centric search with the spectral library using the software DIA-NN,^35^ and uploading of result files back to the openBIS server including quantitative matrices for proteins and peptides.

### Statistical analysis

We will describe patient data as displayed in Table 1. Demographic patient data will be analysed using independent samples *t*-test, Pearson Chi-square test, or Mann-Whitney *U* test as appropriate. Statistical analysis will be performed with the use of R: A Language and Environment for Statistical Computing for the demographic data analysis and Python package “*data processing kitchen sink*” (DPKS) for proteomic analysis.^36^, ^37^

**Table 1:** Example table for clinical characteristics and baseline variables.*

Characteristic	Good outcome *N* =	Poor outcome *N* =	33 °C group *N* =	36 °C group *N* =
Demographic characteristics				
Age in years	Mean ± SD	Mean ± SD	Mean ± SD	Mean ± SD
Male sex	*N* (%)	*N* (%)	*N* (%)	*N* (%)
Characteristics of the cardiac arrest				
Bystander witnessed cardiac arrest	*N* (%)	*N* (%)	*N* (%)	*N* (%)
Shockable rhythm	*N* (%)	*N* (%)	*N* (%)	*N* (%)
Minutes from cardiac arrest to ROSC	Median (IQR)	Median (IQR)	Median (IQR)	Median (IQR)
Clinical characteristics on admission				
First measured body temperature in °C	Mean ± SD	Mean ± SD	Mean ± SD	Mean ± SD
Glasgow Coma Scale score	Median (IQR)	Median (IQR)	Median (IQR)	Median (IQR)
Pupillary reflex bilaterally present	*N* (%)	*N* (%)	*N* (%)	*N* (%)
Serum pH	Mean ± SD	Mean ± SD	Mean ± SD	Mean ± SD
Serum lactate in mmol/liter	Mean ± SD	Mean ± SD	Mean ± SD	Mean ± SD
Circulatory shock	*N* (%)	*N* (%)	*N* (%)	*N* (%)
ST-segment elevation myocardial infarction	*N* (%)	*N* (%)	*N* (%)	*N* (%)
Medical history				
Previous cardiac comorbidity	*N* (%)	*N* (%)	*N* (%)	*N* (%)
Arterial hypertension	*N* (%)	*N* (%)	*N* (%)	*N* (%)
Previous TIA or stroke	*N* (%)	*N* (%)	*N* (%)	*N* (%)
Diabetes mellitus	*N* (%)	*N* (%)	*N* (%)	*N* (%)
Asthma or COPD	*N* (%)	*N* (%)	*N* (%)	*N* (%)
Allocation to 33 °C	*N* (%)	*N* (%)	*N* (%)	*N* (%)
Poor outcome (mRS 4–6) at 6 months	*N* (%)	*N* (%)	*N* (%)	*N* (%)

* Results will be reported as numbers (percentages), median (interquartile range), or mean (±standard deviation) as appropriate. COPD, chronic obstructive pulmonary disease; mRS, Modifed Rankin scale; TIA, transient ischemic attack; ROSC, return of spontaneous circulation.

#### Data filtering

Only charged peptides and proteins passing 1% false discovery rate (FDR) threshold will be considered for downstream data analysis which will be achieved by filtering the data at 1% global precursor *q*-value and 1% global protein *q*-value. For the differential expression analysis, minimum of five samples with valid values per group will be required.

#### Normalisation

To minimize batch effect and technical variation between the samples, normalisation will be applied on charged peptides (precursors) intensities. Normalisation ensures that data from various samples are adjusted to a common scale, simplifying direct comparison. The *normalize()* function in DPKS package^37^ provides a choice of different normalisation algorithms, such as total ion chromatogram, mean, and median sample normalisation, which will allow us to adopt a normalisation method best-fitted for our samples. Option to perform retention time window normalisation is also available.^38^ The final normalisation method will be determined from the alignment of kernel density estimation plots of samples precursor intensities for the samples.

#### Quantification

To infer the precursors to quantified proteins, quantification function of DPKS (*quantify()*) will be used, providing methods for relative quantification (*iq,* TopN).^39^, ^40^ The *iq* implementation of the MaxLFQ algorithm derives optimal inter-sample ratios for each protein, subsequently integrating these ratios into a final protein quantity. The TopN method enhances protein quantification by selecting and aggregating the most intense ions, ensuring robust and accurate relative abundance measurements. A combined quantification approach will be used, where *iq* quantification is applied to the indicated TopN precursors for each protein. This method preserves the protein’s overall abundance ranking while leveraging signal smoothing and ratio extraction techniques for enhanced relative quantification.

#### Exploratory principal component analyses and differential expression analysis

Principal component analyses will be used to perform exploratory analyses of the proteomics data. Furthermore, Uniform Manifold Approximation and Projection will be considered for this type of analysis.^41^ The likely differentially abundant proteins will be extracted using the *compare()* function in the package DPKS, applying a two-sided *t*-test with the method set to “ttest” in combination with multiple testing correction.^37^ We will use the Benjamini Hochberg method to control the FDR. In general, data from three time points, 24, 48, and 72 h after ROSC will be analysed according to the prespecified outcomes and stratifications. Differential protein abundance, expressed as log2 fold change (FC) will be stratified according to survival, good versus poor functional outcome, targeted temperature management of 33 °C and 36 °C, and the MIRACLE2 score. The following TTM-trial design variables may be included in multivariate linear regression analysis to adjust for potential confounding factors and may also serve as secondary stratifications variables in differential protein expression analyses. This is particularly important given the variability in patient characteristics such as sex and age, which, if unaccounted for, could bias the results.

• **Sex** – men vs. women
• **Age** – < 65 or ≥ 65 years.
• **Time to ROSC** – < 25 or ≥ 25 min.
• **Shockable vs. non shockable initial cardiac rhythm**. Defined as shockable if ventricular fibrillation or pulseless ventricular tachycardia was seen as initial rhythm. Defined as non-shockable if asystole or pulseless electrical activity was seen as initial rhythm.
• **Circulatory shock on admission to hospital unit, defined as present versus not present.** Circulatory shock was defined as a systolic blood pressure of less than 90 mmHg for more than 30 min or end-organ hypoperfusion, including cool extremities, urine output of < 30 ml per hour, and a heart rate of < 60 beats per minute.
• **Presumed cause of death.** The presumed cause of death was based on clinical judgment reported by the treating physicians: cardiovascular, cerebral, multi organ failure, other, and undetermined.

Differences in protein regulation according to subgroup will be declared based on statistically significant abundances of proteins in the secondary analyses. Furthermore, machine learning tools may be used to enhance the analyses.

### Data availability

The acquired mass spectrometry data, result files from peptide-centric analyses, spectral libraries, and other metadata will be deposited to the ProteomeXchange Consortium and made publicly available.

## Discussion

In this explorative study, we will use quantitative capabilities of mass spectrometry with diaPASEF method to compare differential protein abundances according to prespecified stratifications in a cohort of cardiac arrest patients. We aim to examine proteomic profiles related to survival, functional outcome, targeted temperature management, and the MIRACLE2 score that may help identify novel biomarkers and/or therapeutic targets. As the largest proteomic study in OHCA patients performed to date, we will include large-size sample data from multiple centres and countries. This will allow better exploration of proteomic patterns among patients and may therefore lead to more accurate comparisons between outcomes. Our study is designed to validate the findings from our pilot study with the aim of revealing prognostic biomarkers, define clinical and proteomic phenotypes of the post-cardiac arrest syndrome, and illuminate the inflammatory and mechanistic trajectory of the syndrome.^42^

Few comparable studies exist for proteomic data in patients with cardiac arrest.^43^, ^44^, ^45^, ^46^ In comparison to the previous studies, our pilot study revealed contrasting proteomic profiles according to functional outcome.^11^ We identified several proteins of interest for further validation as potential biomarkers, such as insulin-like growth factor binding protein 2, vitamin K dependent protein Z, protein Niban 3, kallistatin, and angiotensinogen. In search of novel biomarkers, proteomics aims to identify proteins that are reproducible and consistent in their timely abundance. By repeating the mass spectrometry analysis on the serum samples analysed in the pilot study, we can examine reproducibility patterns of the previously identified proteins. To our knowledge, only one other study to date has examined protein profiles in patients treated with targeted temperature management.^9^ While findings in our pilot study suggest a modest effect of the temperature level on protein abundance, reproduction of these results may further explore proteomic profiles related to targeted temperature management.

Our pilot study could not identify any of the established and proposed biomarkers for cardiac arrest, as they are considered low-abundant proteins. The vast dynamic range of protein concentrations in biological samples and high-abundant proteins can lead to difficulties in identification of low-abundant proteins, necessitating the need for specific enrichment strategies.^19^, ^20^ We will use enrichment strategies in a subset of samples to try identifying the previously suggested neurological biomarkers.^21^, ^22^

### Strengths and limitations

According to our knowledge, this will be the largest proteomic analysis of a cardiac arrest population. Serum samples were prospectively collected during a randomised controlled trial and serial measurements will allow for analyses of changes in protein abundance over time. Conservative approach to neurological prognostication and structured follow-up at 180 days are strengths of the TTM-trial.^8^ Mass spectrometry analysis will be performed using a modern timsTOF HT instrument, providing high dynamic range and analytical depth.^47^ The instrument supports diaPASEF mode, which allows for higher conversion of the incoming ion beam into fragments for peptide identification as compared to DIA.^23^

Sample collection for the TTM-trial was performed 2010–2013 and a subset of samples have undergone at least one freeze–thaw cycle, which may contribute to protein degradation and impact the accuracy of protein quantification.^48^, ^49^ During the trial, temperature control was started within four hours after OHCA (median time approximately 2 h), meaning that temperature control-dependent protein abundance could have been influenced by initiating hypothermia earlier.^8^ The amount of identified proteins may have been altered or limited by usage of serum instead of plasma samples.

## Conclusions

We present the design of a large explorative study examining proteomic profiles in OHCA patients according to survival, functional outcome, targeted temperature management, and the MIRACLE2 score. Our results will provide information about changes in protein abundance first days after cardiac arrest and may help identify novel biomarkers for prognostication of cardiac arrest.

## CRediT authorship contribution statement

**Gabriele Lileikyte:** Writing – review & editing, Writing – original draft, Visualization, Project administration, Methodology, Conceptualization. **Anahita Bakochi:** Writing – review & editing, Supervision, Resources, Project administration, Methodology, Conceptualization. **Marc Isaksson:** Writing – review & editing, Software, Methodology, Conceptualization. **Filip Årman:** Writing – review & editing, Supervision, Software, Resources. **Marion Moseby-Knappe:** Writing – review & editing, Supervision, Resources. **Johan Malmström:** Writing – review & editing, Supervision, Resources, Methodology, Conceptualization. **Niklas Nielsen:** Writing – review & editing, Supervision, Resources, Methodology, Funding acquisition, Conceptualization.

## Funding

Funding for the study was provided by the 10.13039/501100004359Swedish Research Council; Swedish Heart Lung Foundation; Arbetsmarknadens Försäkringsaktiebolag Insurance Foundation; the Skåne University Hospital Foundations; the Gyllenstierna-Krapperup Foundation; Governmental funding of clinical research within the Swedish National Health System; the County Council of Skåne; the 10.13039/501100007687Swedish Society of Medicine; the Koch Foundation; 10.13039/501100007437TrygFonden (Denmark); European Clinical Research Infrastructures Network; Thelma Zoega Foundation; Stig and Ragna Gorthon Foundation; 10.13039/501100016049Thure Carlsson Foundation; Hans-Gabriel and Alice Trolle-Wachtmeister Foundation for Medical Research; the Swedish Brain Foundation; the 10.13039/501100003554Lundbeck Foundation; and the Torsten Söderberg Foundation at the Royal Swedish Academy of Sciences. The funders had no role in study design, data collection and interpretation, or the decision to submit the work for publication.

## Declaration of competing interest

The authors declare that they have no known competing financial interests or personal relationships that could have appeared to influence the work reported in this paper.
